# Revealing the Origin: The Secrets of Textile Fragments Hidden Inside the 19th Century Chasuble from Dubrovnik

**DOI:** 10.3390/ma14164650

**Published:** 2021-08-18

**Authors:** Danijela Jemo, Djurdjica Parac-Osterman

**Affiliations:** 1 Art and Restoration Department, University of Dubrovnik, 20000 Dubrovnik, Croatia; 2 Faculty of Textile Technology, University of Zagreb, 10000 Zagreb, Croatia; dparac@ttf.hr

**Keywords:** natural dyes, SEM-EDX, FTIR-ATR, HPLC, 19th century textile, Dubrovnik

## Abstract

This paper presents the analysis carried out during the textile conservation–restoration process with the goal to reveal the secrets of textile fragments hidden inside the 19th century chasuble from Dubrovnik. The discovered textile fragments were investigated by modern instrumental methods and compared with the original textile from the 19th century set of liturgical vestments, which the chasuble belongs to. In addition, all other old repairs and treatments on the chasuble that had significant impact on the historic textile over time were investigated and assessed. The polymer type of the fibres was established by microscopic examination and infrared (ATR FT-IR) spectroscopy. A comparison of type of fibres and textile construction parameters, both from fragments and from the original textile, was carried out in order to determine their possible associations. Based on UV-Vis and HPLC identification of chemical composition of dyes in extracts from textile fibres, both from textile fragments, old repairs and authentic historic textile, it was possible to designate some common characteristics of dyes as important factors in determining its authenticity.

## 1. Introduction

Archaeological and ancient textiles and their dyes represent valuable sources for the study of clothing and textiles during human history [1,2,3]. Studies on the history of clothing and textiles reveal the religious, social and legal regulations of clothing, as well as their cultural significance and historical, aesthetic or sentimental value [4]. In the absence of valid documentation and origin data, textiles must be defined relying on their specific design and patterns, decoration, fabric types and weaves, dyes, through iconography, modern ethnographic surveys and contemporary scientific analysis [2,4,5].

Based on interdisciplinary approaches and the involvement of specialists from different fields, it is possible to achieve a better understanding of the ancient textiles and their characteristics [6].

The analysis of historic material always involves balancing the usefulness of the information that can be obtained against risk to the integrity of the object. Conservation–restoration treatment is a key to the long-term preservation of textiles. Directions and recommendations given by the European Network for Conservation—Restoration Education (ENCoRE) describe the conservation–restoration process from examination, the highest level of research and diagnosis up to direct intervention or preventive action, if required [6]. Appropriate analytical methodology approach for the analysis of textile fibres and dyes in textile conservation may give a useful indication of the history and origins of a textile, but also how the material has been processed in the past and what methods of conservation treatments to apply in order to preserve it for the future [7].

Each historical item is unique and can tell us its story. Their interesting life span represents a valuable source for different types of case study research. During history, especially the years of the Second World War and after, old textile materials derived from different sources were often employed to make new garments or to repair those that were damaged [2,7,8,9,10]. Worn parts were often cut away, while those which remained preserved were used as patches in repair work. Such is the case with the cultural heritage items from Dubrovnik investigated in this paper.

The Republic of Ragusa (today’s city of Dubrovnik) existed from the 14th to the 19th century. It was an aristocratic, free city-state, with highly developed maritime trade and a diplomatic network. The textile industry of Dubrovnik was closely related to the production of woollen fabrics (*Artis Lane*). Since the production of silk fabrics was poorly represented, in the 19th century, they were mainly imported from the Orient, the Czech lands and England. One of the highly important crafts in the Republic was the dyeing of fabrics (*Artis Tinctoria*), first mentioned in the conclusions of the Grand Council from 1392. Natural dyes of plant and animal origin were used to dye the fabrics. Sources of natural purple-red dyes used in Dubrovnik area from the 14th to the 19th century were brazilwood (*Caesalpinia brasiliensis*), madder (*Rubia tinctoria* L.), kermes (*Kermes vermilio* L.) and Tyrian purple (*Murex trunculus*) [11].

The set of liturgical vestments is one of the five different sets (red, green, black, white and gold color) from the church of the Annunciation of the Blessed Virgin Mary (built in 15/16th century) from the island of Lokrum in front of the city of Dubrovnik. Based on the study of construction technique and distinctive stylistic features, all sets were dated back to the second half of the 19th century [12].

The items are the property of the Diocese of Dubrovnik, but due to the lack of previous documentation, the original owner is unknown. According to the parish priest, it can be assumed that during the 20th century, the sets were brought from the Dubrovnik Cathedral for the purpose of performing a liturgical service on the island. In 2008, with the beginning of the construction works on the island church, they were sent to the restoration workshop at the University of Dubrovnik. The main goal was to make an inventory list with a main documentation. Provided data served as a basis for protecting and preserving that cultural heritage and being put on the list in the Register of Cultural Property of the Republic of Croatia.

**Figure 1:**
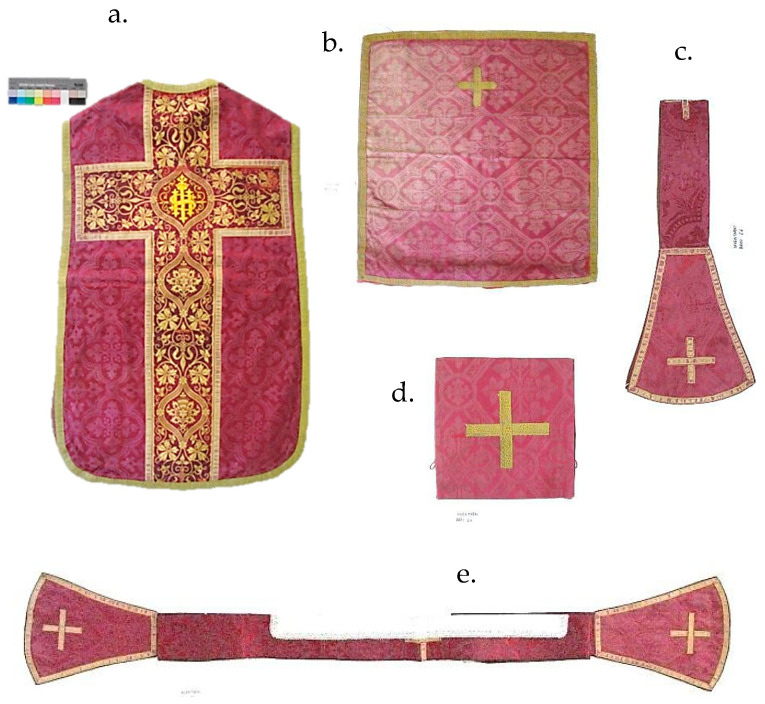
Set of liturgical vestments: (**a**) chasuble, (**b**) velum, (**c**) maniple, (**d**) burse and (**e**) stole.

A case study about the authenticity of subsequent interventions at the 19th century chasuble from Dubrovnik is highlighted in this paper. The chasuble is a part of a set of liturgical vestments that additionally consists of a velum, maniple, burse and stole (Figure 1). Subsequent interventions discovered inside the chasuble include added textile fragments, which are presumed not to belong to the original item, and old stitching repairs done by hand, with yellow and red tone thread, as attempt to arrest or reduce the rate of textile deterioration.

In the “Textile of Parament: Church Textiles of the Croatian History Museum”, Pavičić mentions the fraternity weavers’ workshops linked to the cathedrals alongside the coastal part of the Croatia—in Dubrovnik, Split and Trogir. Their members were knitted and tailored clothes and repaired older church vestments [13]. Some items made in these workshops were composed of various fabrics that originated from different damaged items. Those items previously had a different original purpose or originated from various historic periods, which greatly complicates their dating. Important historical information helpful to date this liturgical set refers to the maniple that a priest once used to wear over his arm. In 1967, the Roman Catholic Church removed the necessity of using a maniple in the liturgy [14].

In order to preserve liturgical sets from Dubrovnik, the undertaking of conservation–restoration treatment was decided. These works were carried out at the University of Dubrovnik. During the conservation–restoration process of the chasuble and velum material, samples were taken and different analyses were performed [12,15,16]. Identification methodology implied the use of non-destructive and micro-destructive testing, using modern instrumentation methods and analysis techniques.

In a combination of physical, biological and/or chemical damaging factors, different deterioration of cultural objects may occur [9,17,18]. Some changes in historic textiles can be caused by manufacturing processes. The process of artificial silk weighting in the late 19th and early 20th centuries can cause rotting and weakening of textile fibres, leading to breakage over time [9]. Additionally, metal salts based on iron used as a dye fixative (mordant) to give a rich dense black colour can deteriorate fibres [9,18,19].

In the middle of the 19 century, synthetic dyes came into the scene and gradually suppressed natural dyes that were exclusively used until then for colouring of textiles [20]. The invention of synthetic dyes had a big impact on methods of dyeing in respect to application, fastness properties, colour range and availability.

Different instrumental techniques and methods of analysis allow us to better understand historical textiles, reducing the rate of textile deterioration and increasing the lifetime of fragile artefacts. The application of scientific methods to the conservation of cultural heritage commenced at the end of the 18th century, and just over a hundred years later they were still restricted to spot tests, optical microscopy and some spectroscopy techniques [21]. However, today modern analytical techniques offer a great deal of information extending the scope of classical, chemical and physical analysis. Physical characteristics of the fibres, such as colour, longitudinal and cross-sectional shape, can be observed with transmitted light [7,22,23]. Infrared microspectroscopy provides complementary information to optical microscopy when identifying polymer type of fibres, but is also ideally suited to the examination of organic materials such as natural dyes [24,25,26]. Day analysis in historical textiles and archaeological finds poses particular challenges, especially as the percentage of dyes are usually much lower compared to recent, contemporary textiles. Scanning electron microscopy (SEM) equipped with an energy-dispersive X-ray spectrometer (EDS) and XRF can provide valuable information about the composition of inorganic aspects, for example, mordant used with the dyestuffs [7,9,27,28]. Until the second half of the 19th century, archaeological and historical textiles were dyed with natural dye sources [3]. High-performance liquid chromatography (HPLC) combined with spectrophotometric UV-Vis detection [29] and mass spectrometric detection [30] is used for characterisation of dyestuffs or pigments that may have been used as colorants in historical textile materials.

Principles of textile conservation involve systematic study, documentation and scientific investigation of textiles. To offer a more complete understanding of each textile artefact that is unique and irreplaceable, it is necessary to determine its construction, materials, condition and authenticity [31].

Analytical methodology applied in this work was adapted to the specific demands that arise when cultural heritage material is analysed. The primary aim of this study was the application of scientific methods to the conservation–restoration of cultural heritage that brings a new dimension to the understanding of a historical object. Supplemented by the comparative analysis, it was a useful tool to explain differences and similarities between the original historic textile and the fragments, revealing the hidden secrets. Identification of the chemical composition of dyes gave valuable data about the cultural heritage textile located in the area of Dubrovnik.

## 2. Materials and Methods

### 2.1. Materials

In textile conservation, material investigation carried out on textile samples of original artefacts consider taking small amount of all different types of warp and weft, since they may contain different component materials [9,32].

To identify and compare fibre content and present dyes, a representative amount of warp and weft thread samples of the original historic textile was taken from the chasuble and velum item. The samples of threads from the textile fragments and stitches on chasuble were also taken. Considering the value and the fragility of cultural heritage material, the threads were cut away from the hidden parts or mechanically damaged areas without damaging the integrity of the object.

For each instrumental method applied in this work, up to 5 millimetres of thread were cut away from the warp (*Sample 1, 3, 5, 7*, Table 1) and the weft (*Sample 2, 4, 6, 8*, Table 1), as well as from the stitches (*Sample 9 and 10*, Table 1). The optimum sample length used for the investigation is determined based on the satisfactory results obtained by testing such a small amount of sample, respecting the fact that the cultural heritage is explored. Based on microscopic observations and the information about the general structure of the fibre obtained from the surface, we conclude that the quality of the samples is satisfactory to provide enough evidence for the identification [15].

#### 2.1.1. Original Historic Textile

The set of Liturgical vestments from Dubrovnik is composed of five different pieces, made from various textile materials (Figure 1).

Based on the previous visual and tactile analysis, we came to the conclusion that all other items of the set are made of the same red damask materials found on the chasuble (Figure 1a) and velum (Figure 1b) [15]. Therefore, it was enough to take only samples of damask fabric from the chasuble and velum for the analysis (Table 1a,b).

The examination of samples under an optical microscope revealed that fabrics were made of two types of fibre. It is not a mixture of fibres, but a weave structure where all warp samples are 100% silk; the same applies to the weft samples taken from all fabrics, which are 100% cotton. This was confirmed by FTIR-ATR analysis that gave a high percentage of matching with the reference fibre, further discussed in Section 3.1.1.

#### 2.1.2. Old Repairs on Chasuble—Textile Fragments and Stitches

In the condition report, based on a close examination of the chasuble, various damages were noted such as surface impurities, stains and damage caused by wear and tear, deformation of the material, damages in the weave structure, loss of seam integrity on the stitch line joining two pieces of fabric, discoloration, folds, creases and previous repairs [15].

There are also subsequent interventions present that appeared over time, such as red damask fragments (Table 1c,d) and repairs by sewing with red and yellow threads (Table 1e). During conservation and restoration works, damask fragments were found hidden inside the chasuble (Table 1f). They are located on the backside of the object, under the lampas fabric (Table 1c), as well as a small fragment attached to the interlining inside the chasuble and stitched with red thread (Table 1d). Discovered fragments beneath the cross on the backside of the chasuble are used to support damaged lampas fabric, distributing the weight of the damaged textile evenly. The true meaning and role of Fragment 2 will become understandable only later, after the analysis. Sample descriptions taken from Fragment 1 and Fragment 2 are given in Table 1.

Stitches that have been sewn by red and yellow thread, designated as old repairs or subsequent interventions on the chasuble, are given in Table 1e. These stitches appeared over time during use, found on damaged parts of the original damask and lampas fabric. The first one is dense lines of yellow stitching which overlay the surface of lampas fabric, following the yellow weave pattern. The second repair is the red thread of the support stitch, whose role was to securely anchor the lost weft threads from damask fabric. The same red thread stitch was found in the lampas fabric and in Fragment 2 (Table 1d).

### 2.2. Methods

**Figure 2:**
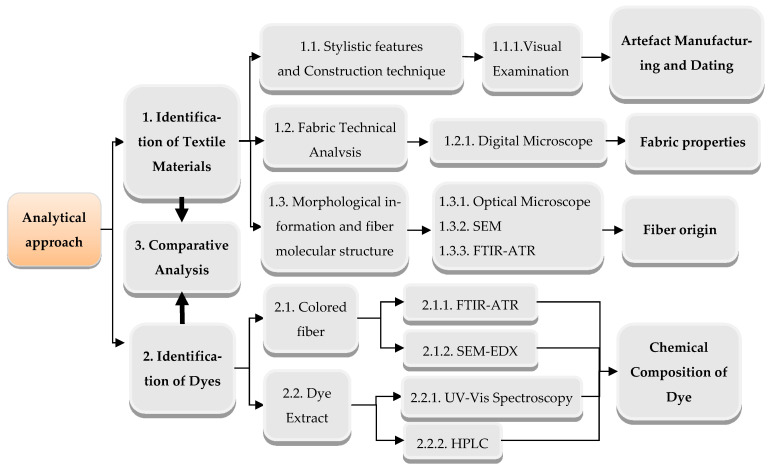
Analytical Methodology Applied.

Performing the research according to the algorithm shown in Figure 2, each technique provided different data useful for analysis and identification purposes. Yarns or threads from the textile fabrics or seems, as well as the dye extract from those samples, were investigated.

Visual analysis of the items’ construction, such as cutting parts and structural seams, gave valuable information about the shape of the artefact, production and style, relevant for the typological dating. Fabric design and patterns revealed the fabric type characteristic of the specific historical period (Figure 2, block 1.1).

Using a digital microscope, fabric technical analysis was performed, obtaining data about the fabric properties, such as weave structure and type, yarn twist and fabric density. This information was important for the historic textile documentation, as well as for the comparative analysis (Figure 2, block 1.2).

Different techniques and data science tools were used for comparing and classifying textile fibres (Figure 2, block 1.3). Optical microscopy was used to examine each sample in order to verify that the samples are homogeneous and do not contain any other fibre types (Figure 2, block 1.3.1). The polymer type of the fibres was established by optical microscopy, SEM and FTIR-ATR (Figure 2, blocks 1.3.1, 1.3.2, 1.3.3).

The identification of the dye components present on the coloured fibres was performed by FTIR-ATR, SEM-EDS, HPLC and UV-Vis Spectroscopy, on both the solid samples and dye extracts (Figure 2, blocks 2.1 and 2.2). The analysis provided data on the chemical composition of each dye present in the sample to confirm dye sources used as colorants on the investigated historical textile materials.

Finally, a comparative analysis was applied to identify and match textile fabrics, yarns, fibres and dyes in order to confirm their authenticity and to establish common features (Figure 2, block 3).

Abbreviations: SEM, (Scanning electron microscope), FTIR-ATR (Fourier-transform infrared spectrometry with attenuated total reflectance), SEM-EDX (Scanning electron microscopy with an energy dispersive X-ray Spectroscopy), HPLC (High performance liquid chromatography).

#### 2.2.1. Instrumentation and Software

A preliminary examination using digital microscope Dino-Lite Pro AM 413T (1.3 megapixel image sensor, 10×−50×, 200× magnification, 8 White LEDs On/Off, USB 2.0 output and software Dino Capture 2.0. (Almere, The Netherlands) provided a quick, accurate and non-destructive method to characterise textile surface material (weave structure, thread count, surface damage).

The polymer type of fibres was found by microscopic examination and infrared (IR) spectroscopy. Optical microscope analysis provided clear views of the morphology of the fibres. All fibre samples are examined with an Olympus BX40F4 microscope (ocular WH10x/22, objectives 10× and 40×) connecting to the computer via Olympus SC30 camera 3.3 MP (2048 × 1532 resolution, Olympus Europa SE & Co. KG, Hamburg, Germany) and software Stream Start (v1.5.1).

Advantages of using scanning electron microscopy (SEM) were the ability to visually characterise fibres plus elemental analysis to identify mordant present in the samples. The samples were investigated using a TESCAN Scanning Electron Microscope (FE-SEM, MIRA\LMU, Czech Republic, program Mira TC, In-Flight Beam TracingTM, EasySEMTM, Wide Field OpticsTM) equipped with energy-dispersive spectroscopy (EDX with detector Quantax, Bruker SC7620-CF Mini AXS Microanalysis).

FTIR spectroscopy (FTIR-ATR, PerkinElmer Spectrum 100) was applied to confirm whether the characteristic peaks for the suspected organic polymers were present in the spectra of the sample, using the spectra of known fibres as reference. FTIR-ATR was also used for identification of dye components present in colored fibres. FTIR-ATR of textile samples was traced in four scans at a resolution of 4 cm^−1^ in a wavelength range from 4000 to 400 cm^−1^ and software *OMNIC* *Spectra* Software *9.1.24*. By using the same identical scan conditions to record both the fibre (reference) spectrum and the spectrum of the coloured sample, the intensity of the raw fibre spectrum was removed from the sample spectra by digital subtraction. Collected spectra were subtracted using *Omnic 9.1.24* software, thus gaining a better understanding of the dye component.

The chemical constituents of the extracted dye (Section 2.2.2) were identified through spectrophotometric and chromatographic analysis. The extracted dye was dissolved using water as solvent and scanned through UV-Visible spectrophotometer (Varian Cary^®^ 50, Shimadzu Europe, Duisburg, Germany). The wavelength of the dye at lambda (k) max was measured and the compounds present in the extracts were interpreted.

HPLC analysis of dyes were carried out using an Agilent 1200 series system (Agilent Technologies, Hewlett-Packard, Germany), including a diode array detector and the chromatographic system controlled by *ChemStation* software. HPLC conditions: C18 column 4.6 × 150 mm, 5 nm (4.6 × 150 mm and 5 μm particle size, ZORBAX Eclipse XDB, mobile phase A = 10% of methanol/water, *v*/*v*, B = 100% of methanol, gradient at start 16% B, at 15 min 90% B, at 23 min 100% B and 30 min 60% B, flow rate 0.5 mL/min, temp. 25 °C, injection vol. 10 mL, identification wavelength 254 nm. All reagents were analytical grade: methanol (HPLC-hipergradient grade), dimethylform-amide DMF (HPLC-gradient grade), formic acid (HPLC-gradient grade) from Merck, Germany. The method used to identify the dye was that of Jemo and Parac (2017).

All additional data of the equipment used in this research are described in the Equipment catalogue of the University of Zagreb Faculty of Textile Technology [33].

Literature sources and online databases were used as a database of natural colours for FTIR analysis (IRUG-Infrared and Raman Spectral Database Users Group: Schweppe Collection, Getty Conservation Institute, [34]; SDBS-Spectral Database for Organic Compounds, [35]) and for HPLC analysis, (Eu-ARTECH Project-Analytical strategies for natural dyestuffs and cultural heritage objects, [30,32,36,37,38,39,40,41,42,43,44,45,46,47,48,49,50].

#### 2.2.2. Extraction of Colorant

The dye was extracted from both warp and weft textile samples (~0.002 g, fragments and original textile) using a solution of 5% Formic acid/MeOH at 100 °C for 10 min. Then, the solution was filtered (Chromafil PET-45/25, Macherey-Nagel, Düren, Germany) and a volume of 250 μL of the mixture MeOH; DMF (1:1, *v*/*v*) was added to the dry residue, then heated for 5 min at 100 °C. The extract obtained was used for UV-Vis i HPLC analysis.

## 3. Results and Discussions

The starting point for making decisions in the textile conservation process to discover the materials present in any object is an examination [18]. It is equally important to analyse the original material, as well as the one added during a later time. Analytical techniques suitable for the analysis of cultural heritage are highly complex, and extremely small amounts of fibre sample are allowed to be taken from the carefully selected area. That is why the identification of textile materials was performed first, followed by the chemical composition of dyes, as shown in Figure 2.

### 3.1. Identification of Textile Materials

Analyses were carried out on warp and weft samples derived from the original textile fabric (Chasuble and Velum, Table 1a,b), from fragments (Fragment 1 and 2, Table 1c,d) as well as from sewing threads of old repairs (Table 1e).

Optical, scanning electron microscopy and FTIR-ATR analysis was used for fibre identification, while a Dino-Lite digital microscope with magnification: 10×−70×, 200× was used for weave and yarn characterisation.

#### 3.1.1. Fibre Classification

**Figure 3:**
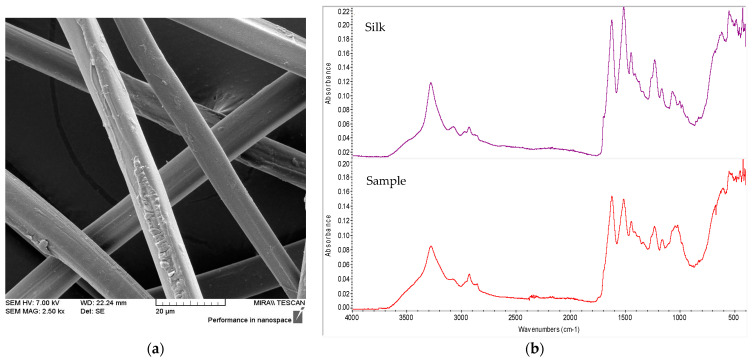
Silk warp from Fragment 2, *Sample 7*: (**a**) SEM photograph; (**b**) FTIR-ATR spectra of reference silk and sample (red).

The surface morphology of all fibres was investigated using optical and scanning electron microscopy. It was found that analysed warp samples (*Sample 1, 3, 5* and *7*; Table 1a–d) correspond to the protein-based silk fibres (Bombyx mori, Figure 3a), which have also been confirmed by FTIR-ATR analysis (Figure 3b). Using OMNIC Spectra Software, the statistical discriminant analysis was performed determining that the similarity percentage of the silk fibre spectra from the database compared to each fibre from the warp sample is around 70%.

It should be noted that investigated samples contain other additives in the fibre, such as dyes, impurities, microorganisms and the products resulting from their metabolic activity, which caused a small deviation from the uncoloured reference fibre.

**Figure 4:**
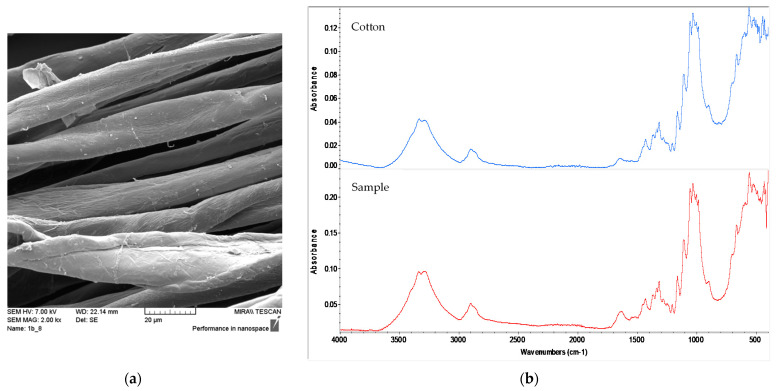
Cotton weft from Fragment 2, *sample 8*: (**a**) SEM photograph; (**b**) FTIR-ATR spectra of reference cotton and sample (red).

On the other hand, all fibres from weft samples (*Sample 2, 4, 6* and *8*; Table 1a–d) exhibited a distinctively flat “ribbon”, with a twist across the fiber length and a kidney-shaped cross-sectional structure, all of which are characteristic of cellulosic seed fibres such as cotton (Figure 4a). Characteristic spectral bands for cotton have also been confirmed by FTIR-ATR spectroscopy (Figure 4b), with the average similarity to the reference cotton fibre of 70%.

Samples of warp and weft recorded by SEM and FTIR are given in Figure 3 and Figure 4.

Due to their organic nature, textiles are among the most sensitive objects of cultural heritage and therefore over time become more fragile [9,17,18]. Damaged textile is often preserved adding repair patches or stitches, more or less successfully.

Repair stitches recorded on the chasuble mutually differ. *Sample 9* (red thread) proved to be mercerised cotton, while *Sample 10* taken from the yellow thread was made of viscous fibre, based on morphological features and confirmed by IR microscopy with the average similarity to the reference fibre of 70%.

#### 3.1.2. Evaluation and Comparison of Fabrics Construction Parameters and Old Repairs

Analysis of fabric construction parameters was carried out with the goal to show differences or confirm similarities between discovered textile fragments hidden inside the chasuble and the original textile from the 19th century set of liturgical vestments from Dubrovnik, as shown in Table 2.

Although all the warp samples (*Sample 1, 3, 5* and *7*) are classified as silk fibre and those from the weft (*Sample 2, 4, 6* and *8*) as cotton, some design and construction fabric parameters differ from each other (Table 2). From Table 2, it is evident that fragments from chasuble and damask from velum share common characteristics of weave pattern and yarn density, but not damask from the chasuble.

Similarities in the samples of warp and weft taken from the textile fragments (Fragment 1 and 2) and damask from the chasuble are observed in colour tone (red tone), type of colorant (Table 4) and raw material composition (silk warp and cotton weft, Table 2), as confirmed by the microscopic, spectroscopic and chromatographic analysis. Nevertheless, the design motive that is identical in both Fragment 1 and 2 differs from the original damask fabric of the chasuble.

In addition, all other old repairs as interventive treatment on the chasuble that had significant impact on the historic textile over time were investigated and assessed. Particularly interesting is the facts that on Fragment 2 (Table 1d) there are identical repairs, made with the same thread and sewing techniques, as well as on lampas fabric (Table 1e, *Sample 9*).

### 3.2. Chemical Composition of Dyes Extracted from Textile Samples

Liturgical vestments from Dubrovnik date to the 19th century, the time when only natural dyes were used. Therefore, the presence of natural dyes, with the exception of subsequent interventions, is expected on all samples in this paper.

Based on the available literature and historical and archaeological research, natural dyes of plant and animal origin, used in Dubrovnik and its surroundings, for dyeing red or purple are: *kermes vermilio* L., *Caesalpinia echinata* L., *Rubia tinctorum* L., *Murex trunculus*, *Dactylopius coccus* costa [45,46,47,48,49,50].

All samples taken from original textile fabrics (chasuble and velum) and from old repairs (textile fragments and sewing threads) were analysed for chemical and functional group identification of their dye components and the presence of mordants.

**Figure 5:**
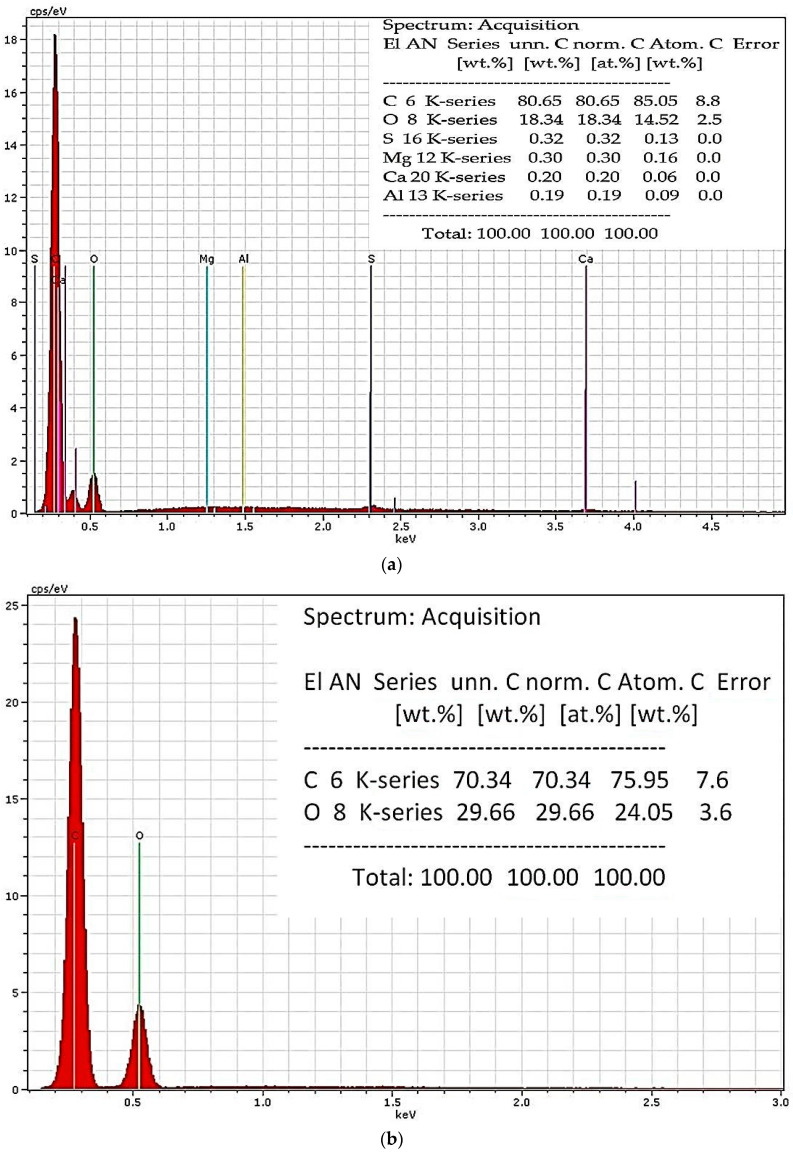
SEM-EDX analysis: (**a**) *Sample 1*; (**b**) *Sample 2*.

Most of the natural dyes are mordant dyes. A mordant is a metallic oxide, which, depending on the chemical constitution, can give different hue colours with the same dye. By using SEM-EDX analysis, it was possible to determine various mordants in coloured samples. Figure 5 shows warp and weft samples from the chasuble damask fabric.

Aluminum detected on the silk warp samples (*Sample 1*, Figure 5a; *Sample 3, 5, 7*) may indicate the presence of inorganic salt, called potassium aluminum sulfate (AlK(SO_4_)_2_), an important historic mordant used in dyeing. On the other hand, there were no elements that could be traced to mordants on the weft cotton samples (*Sample 2*, Figure 5b; *Sample 4, 6, 8*) as well as on *Sample 9* and *10*.

**Figure 6:**
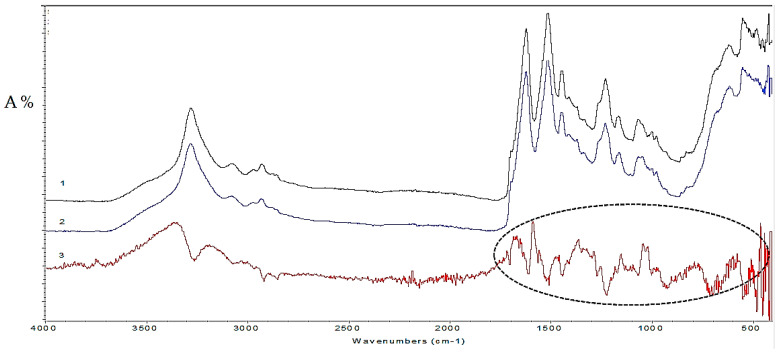
FTIR-ATR absorption spectra: 1-undyed sample of reference silk fibre, 2-*Sample 1*, 3-dye spectrum obtained by spectral subtraction of reference silk spectra from the coloured sample spectrum.

The FTIR-ATR technique did enable the comparison of different samples and characterisation of the polymer, but gave incomplete information on the dyes present in coloured fibre. In order to enhance this information, Omnic 9.1.24 software is used to subtract the spectrum of the characteristic uncoloured reference fibre from the spectrum of the coloured sample, thus making it easier for some individual dye components of coloured samples to be distinguished (Figure 6).

It can be stated that all FTIR-ATR samples spectra from the chasuble highlight absorption bands of 1710 cm^−1^ to 1550 cm^−1^ in which intervals are detected: conjugated C=O groups, carbonyl groups, stretching of C-N bonds, stretching of C=C alkenes and amine N-H stretch. Additionally, absorbing bands of 1450 cm^−1^ to 600 cm^−1^ are characteristic for aromatic compounds, esters, ethers, phenols, alcohols, which are otherwise found in natural dye.

FTIR-ATR analysis of all warp samples (*Sample 1, 3, 5, 7*) shows characteristic fingerprint spectral features of C=C bond stretching, conjugated C=O groups, carbonyl groups, -OH groups in the region of 1100 cm^−1^ to 1600 cm^−1^, which, according to the spectral database (Section 2.2.1) are characteristic functional groups found in madder root.

On the other hand, all weft samples (*Sample 2, 4, 6, 8*) have conjugated C=O groups at 1665 cm^−1^ and stretching of aromatic C=C bond at 1584 cm^−1^ and 1467 cm^−1^, all characteristic of the *Murex trunculus* used in the Dubrovnik area. In addition to its two smaller peaks at 977 cm^−1^ and 947 cm^−1^, this can be attributed to 6,6′-dibromoindigo.

**Figure 7:**
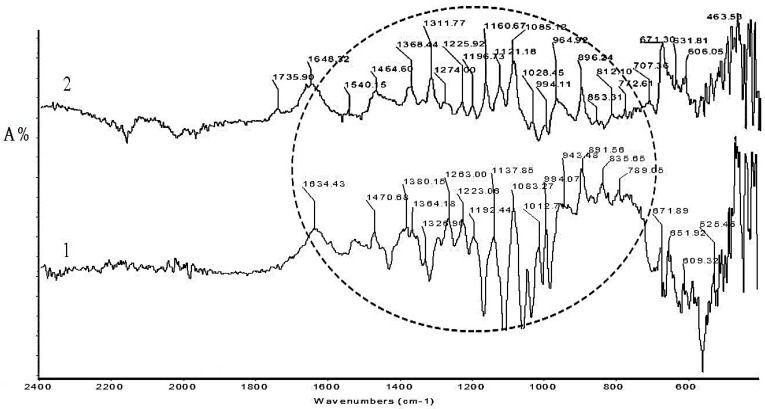
FTIR-ATR absorption spectra: 1-*Sample 9*, 2-*Sample 10*.

Both warp and weft samples exhibit strong peaks in the region of 1100 cm^−1^ to 1700 cm^−1^, corresponding to the stretching vibration of the C=C bond, conjugated C=O groups, carbonyl groups and -OH groups, characteristic for infrared spectra of cochineal dye. The sharp peaks observed at 1700–800 cm^−1^ for *Sample 9* and *Sample 10* are characteristic of synthetic dyes (Figure 7).

Bearing in mind the fact that *Sample 10* (viscose fibre) and *Sample 9* (mercerized cotton) are made of fibres whose production started at the beginning of the last century, it can be concluded that these subsequent interventions occurred during the 20th century.

Chemical characterisation of dyes extracted from warp and weft samples was performed through spectrophotometric and chromatographic measurements. Although UV-Vis absorption spectra from dye extract determine only the physical characteristics of the dye, and achieved spectra can be interpreted at the level of qualitative information, identification of the peaks produced in the sample extracts gave a good indication for further analysis. It is important to note that UV-Vis spectra taken of the whole sample can contain some overlapping data (if more than one dye is present), and therefore the absorption maxima of the UV-Vis spectral profile of the individual dye molecules is not always clearly distinguished. Nevertheless, it can be concluded that Vis spectra of all silk warp samples are slightly different, but they all exhibit an intense absorption band around 500–555 nm, that may indicate their antraquinon origin (Figure 7), as pointed by FTIR-ATR analysis.

**Figure 8:**
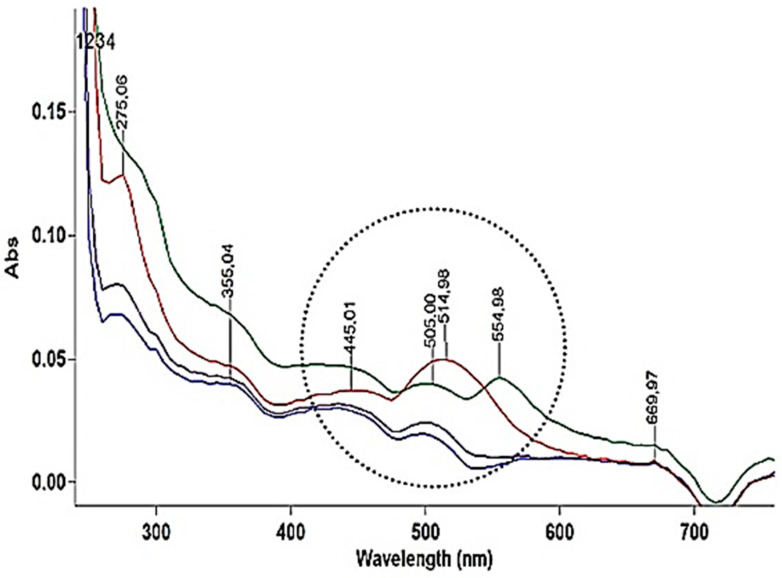
UV-Vis absorption spectrum of cotton wefts: 1-Sample 6 (Fragment 1), 2-Sample 8 (Fragment 2), 3-Sample 2 (Chasuble), 4-Sample 4 (Velum).

Vis spectra of all cotton weft samples show some similarities and intense absorption bands in the region around 500–550 nm (Figure 8).

According to the sample database of original components, the characteristic wavelength of maximum absorption (λ_max_) in the range of 540–555 nm can be attributed to pseudopurpurine, purpurine and colour from the sea snails in the *Muricidae* family, while λ_max_ 510 nm can refer to alizarin, cochineal or kermes dye.

**Figure 9:**
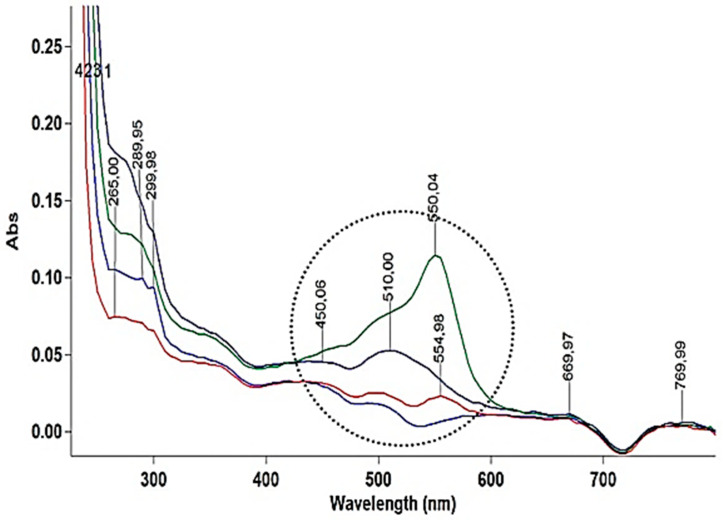
UV-Vis absorption spectrum of silk warps: 1-Sample 5 (Fragment 1), 2-Sample 7 (Fragment 2), 3-Sample 1 (Chasuble), 4-Sample 3 (Velum).

Differences can be observed in the UV area. While the extract of the weft cotton samples (Figure 8) shows λ_max_ at 275 nm, the extract of warp samples (Figure 9) shows λ_max_ at 265 nm and two peaks at 289 nm and 299 nm, indicating that it is a dye from another source.

**Figure 10:**
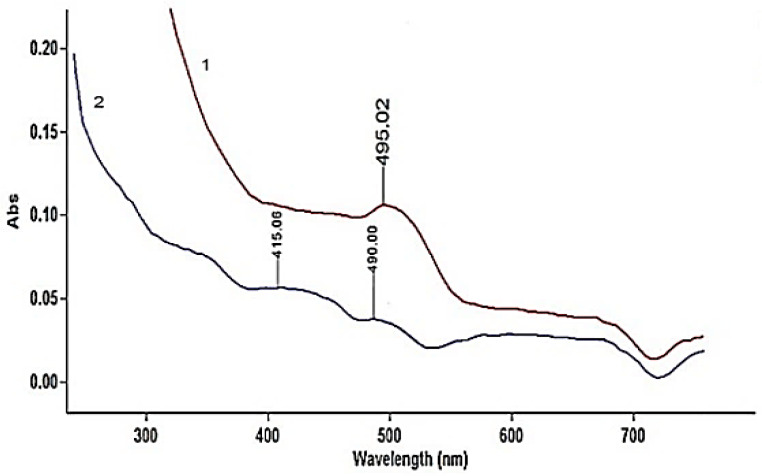
UV-Vis absorption spectra of samples from subsequent sewing interventions: 1-*Sample 9*, 2-*Sample 10* (Velum).

A peak of yellow orange tone λ_max_ 415 and 495 nm characterises *Sample 9* and *10* of the subsequent interventions (Figure 10).

High-performance liquid chromatography (HPLC) was used to identify detectable traces of dye components on extracts from textile samples (Table 3).

**Figure 11:**
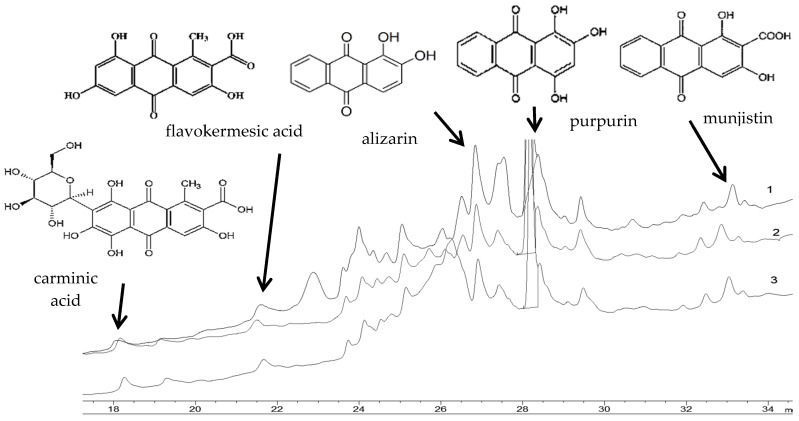
HPLC chromatograms of silk warps: 1-*Sample 1*, from chasuble, 2-*Sample 7*, from Fragment 2 as prior intervention, 3-*Sample 3* from Velum.

Results indicate that the most dominant identified colorants in *Sample 1* (silk warp) are anthraquinones alizarin and purpurin. In addition, a peak at 33 min is attributed to pseudopurpurin or munjistin, all derived from the root cultures of *Rubia tinctorum* L. (Table 3, Figure 11). During the dyeing process, alizarin forms a complex with metal ions such as aluminium, which is identified by SEM-EDX analysis (Figure 5). However, two more peaks were observed at lower retention times in the HPLC chromatogram: carminic acid (rt = 18 min) and flavokermesic acid (rt = 21 min) (Table 3, Figure 11). This result confirms that the dye used in the *Sample 1* is the madder and cochineal dyes.

HPLC chromatograms provide similar values for *Sample 3* (silk warp of the original damask fabric of the Velum) and *Sample 7* (silk warp from fragment 2). FTIR-ATR analysis revealed that those samples have identical infrared spectra as well. Based on the HPLC peak, areas for the three components of the madder dye are identified as follows: alizarin (rt = 26.85 min), purpurin (rt = 28.15 min) and pseudopurpurin or munjistin (rt = 33 min). Additional retention times were also observed, such as ~18 min of carminic acid and ~21 min of flavokermesic acid. HPLC results indicate that the dye used in these samples is a mixture of madder and cochineal dye.

**Figure 12:**
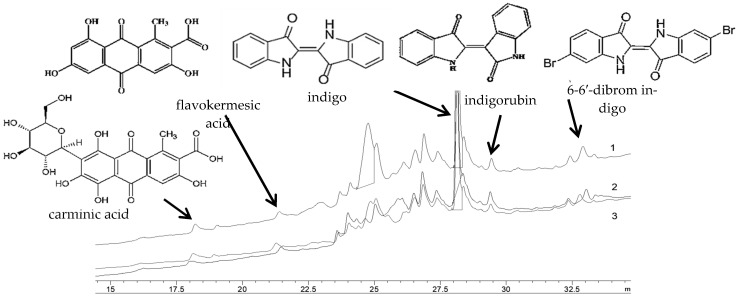
HPLC chromatograms of cotton wefts: 1-*Sample 2*, from chasuble; 2-*Sample 8*, from Fragment 2 as prior intervention; 3-*Sample 4* from velum.

For weft cotton samples (*Sample 2, 4, 6* and *8*), the analytical method with a reducing agent was used to prove the characteristic behaviour of indigoid dyes. Treatment with a reducing agent yielded yellow-green “leuco indigo” form, which then changed to a bluish tone by the processes of oxidation. The major chromatographic peaks seen in those samples showed characteristic retention times at 28.2 min of indigotin, at 29.40 min of indigorubin and at ~33 min of 6,6′-dibromindigo, all characteristic for the Murex sea snail dye (Figure 12). The peak eluted at 18 min was marked as carminic acid and the peak at 21 min as flavokermesic acid (Figure 12). Based on the obtained result and data available in the literature, those compounds indicate the presence of the cochineal dye. Considering the fact that those weft cotton samples contain all chromophore groups mentioned above, it is confirmed that the extracted dye is a mixture of dyes from and cochineal.

The results of dyestuff analysis of investigated samples are given in Table 4.

## 4. Conclusions

Analysis shows that textile fragments discovered inside the chasuble do not belong to the original item. However, they are identical to the fabric that the velum is made of, since they have the same fabric characteristics, the warp and weft are composed of the same type of fibre and they are dyed with the same type of dyes (Table 2 and Table 4).

The right answer to the question of how the textile fragments identical to the velum fabric got inside the chasuble may be is that they come from another set of liturgical vestments that were destroyed over time and then, at one point, used to stabilise the damaged parts of the chasuble in question.

Subsequent repairs of the lampas fabric on the chasuble executed in red and yellow thread are particularly interesting (Table 1e). The same type of red seam is also found in Fragment 2 (Table 1d). Although analysis showed that the Fragment 2 fabric is identical to the damask from the velum, therefore of the same age, the results of the analysis of the sewing thread cast a different light on those repairs. Based on the discovery that fibres are composed of mercerised cotton dyed with the synthetic dye (Table 4), it was concluded that the thread used to make the stitches on fragment 2 is of more recent date.

Given the fact that the stitching thread and the type of stitches found on Fragment 2 are identical to those from the red part of the lampas fabric of the chasuble, it can be concluded that the small, partially damaged parts of the original textile, were used as a test sample to determine the most suitable sewing techniques that were to be applied for the repair of the entire chasuble. For an unknown reason, it was decided to attach the mentioned fragment onto the interlining fabric inside the chasuble in order to preserve it as evidence. In addition, a synthetic dye was identified on a yellow thread from seams, which in regular, thick stitches cover damaged areas on the *IHS* monogram on lampas fabric. This evidence confirms the original thesis that at one point in time the chasuble was separated into pieces in order to carry out its complete stabilisation, after which it was reassembled.

All of the above may suggest that this is a case of repairing worn-out liturgical vestments by removing damaged materials and joining the remaining parts, possibly from two different sets, into one. This assumption is based on the fact that some materials from the chasuble can be found on a few of the other artefacts that make up the liturgical set.

A case study of textile fragments hidden inside the 19th century chasuble from Dubrovnik emphasised the importance of the application of modern instrumentation methods in the textile conservation–restoration study. Forensic scientists often need to identify and match textile fibres using sophisticated scientific instrumentation. The same method was applied in this paper where the fibre evidence and dyes from the fragments were compared with those from the liturgical set.

As assumed, natural dyes were confirmed in all samples from the historic textile, with the exception of subsequent interventions. The red tone silk warps were dyed with two different natural dyes: madder (*Rubia tinctorum* L.) and cochineal (*Dactylopius coccus*); the red tone cotton wefts were also a mixture of the two dyes, dyed with Tyrian purple (*Murex trunculus*) and cochineal (*Dactylopius coccus*). The dyes identified on the historical textiles largely correspond to the known dyes used at that time in the Dubrovnik area.

The results of dyestuff analysis can serve as a basis in creating a database of dyes used in the objects from this area. That will help to better understand the history and origins of each textile artefact that is registered as cultural property under protection by the Ministry of Culture.

## Figures and Tables

**Table 1:** Samples taken from the main fabric of chasuble and velum.

**(a) Chasuble Damask Fabric (Figure 1a)**		
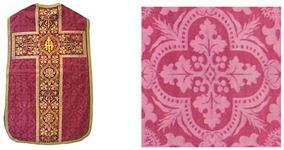	*Sample 1*	red tone silk warp from damask fabric; multifilament yarn spun in the s-direction
	*Sample 2*	red tone cotton weft; 2 s-spun yarns plied in the z-direction
**(b) Velum Damask Fabric (Figure 1b)**		
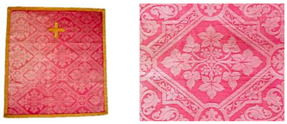	*Sample 3*	red tone silk warp from damask fabric; multifilament yarn spun in the s-direction
	*Sample 4*	red tone cotton weft; z-spun
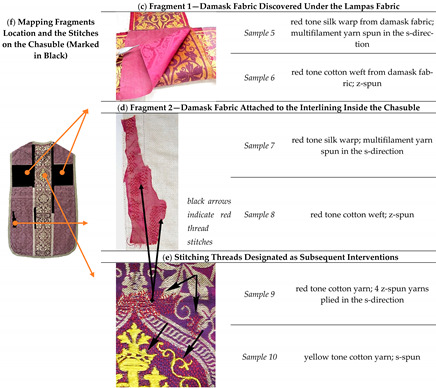		

**Table 2:** Fabrics construction parameters and fibre classification.

Material	Type of Weave	Fabric Density (Threads/cm)		Fibre Identification	
		Warp *	Weft **	Warp *	Weft **
Chasuble	Classic damask in five-end satin, (tal. *Damasco classico*) ^1^	114	28	Silk	Cotton
Velum	Damask woven in combination of eight-end satin and weft-faced twill (tal. *Damasco di Lione*)	64	36	Silk	Cotton
Fragment 1	Damask woven in combination of eight-end satin and weft-faced twill (tal. *Damasco di Lione*)	64	36	Silk	Cotton
Fragment 2	Damask woven in combination of eight-end satin and weft-faced twill (tal. *Damasco di Lione*)	64	36	Silk	Cotton

^1^ The same fabric can be found on the maniple from the same set (Figure 1c) [16]. * *Samples: 1, 3, 5, 7.* ** *Samples: 2, 4, 6, 8*.

**Table 3:** Traces of dye components on extracts from textile samples determined by HPLC chromatography.

High-Performance Liquid Chromatography		
Sample	Rt. Time (min)	Compound
*Sample 1*	26.84	alizarin
	28.14	purpurin
	33	pseudopurpurin or munjistin
	18	carminic acid
	21	flavokermesic acid
*Samples 3, 5, 7*	26.85	alizarine
	28.15	purpurine
	33	pseudopurpurin or munjistin
	18	carminic acid
	21	flavokermesic acid
*Samples 2, 4*	28.2	indigotin
	29.40	indigorubin
	33	dibromindigo
*Samples 6, 8*	18	carminic acid
	21	flavokermesic acid

**Table 4:** Characterisation of dyes of investigating samples.

Material	Sample	Identified Dyes
Chasuble	*Sample 1*	Madder (*Rubia tinctorum* L.), Cochineal (*Dactylopius coccus*)
	*Sample 2*	Tyrian purple (*Murex trunculus*), Cochineal (*Dactylopius coccus*)
Velum	*Sample 3*	Madder (*Rubia tinctorum* L.), Cochineal (*Dactylopius coccus*)
	*Sample 4*	Tyrian purple (*Murex trunculus*), Cochineal (*Dactylopius coccus*)
Fragment 1	*Sample 5*	Madder (*Rubia tinctorum* L.), Cochineal (*Dactylopius coccus*)
	*Sample 6*	Tyrian purple (*Murex trunculus*), Cochineal (*Dactylopius coccus*)
Fragment 2	*Sample 7*	Madder (*Rubia tinctorum* L.), Cochineal (*Dactylopius coccus*)
	*Sample 8*	Tyrian purple (*Murex trunculus*), Cochineal (*Dactylopius coccus*)
Old repairs with stitches	*Sample 9*	Synthetic dye
	*Sample 10*	Synthetic dye

## Data Availability

We have chosen to exclude this statement.
